# Cross-cultural adaptation and analysis of psychometric properties of the Sinhala version of the Attitudes to Aging Questionnaire for institutionalized older adults

**DOI:** 10.1186/s40359-025-03919-y

**Published:** 2025-12-30

**Authors:** Udalamatta Gamage Nirmala Priyadarshani, Ilankoon Mudiyanselage Prasanthi Sumudrika Ilankoon, Sudath Shirley Pathmasiri Warnakulasuriya, Christine Sampatha Evangeline Goonewardena

**Affiliations:** 1 Department of Clinical Nursing, Faculty of Nursing, University of Colombo, Thalapathpitiya, Nugegoda, Sri Lanka; 2 Faculty of Graduate Studies, University of Sri Jayewardenepura, Gangodawila, Nugegoda, Sri Lanka; 3 Department of Nursing and Midwifery, Faculty of Allied Health Sciences, University of Sri Jayewardenepura, Gangodawila, Nugegoda, Sri Lanka; 4 Department of Community Medicine, Faculty of Medical Sciences, University of Sri Jayewardenepura, Gangodawila, Nugegoda, Sri Lanka; 5 Non-Communicable Diseases Research Centre, Faculty of Medical Sciences, University of Sri Jayewardenepura, Gangodawila, Nugegoda, Sri Lanka

**Keywords:** Attitudes to aging questionnaire, Older adults, Sri Lanka, Validation

## Abstract

**Background:**

Attitudes towards aging are multidimensional. It is often affected by the individual’s social and cultural background. Valid and reliable tools across diverse cultures are important to assess the attitudes towards aging among older adults.

**Methods:**

The Sinhala version of the Attitudes to Aging Questionnaire (AAQ) was cross-culturally validated following standard procedures. A validation study was conducted with a sample of 185 Sinhala-speaking older adults residing in residential care facility homes in the Colombo district. The Sinhala version of the AAQ was interviewer-administered along with the World Health Organization Quality of Life-BREF (WHOQOL-BREF) questionnaire. Floor and ceiling effects were assessed for each AAQ subscale, considering effects present if ≥ 15% of participants achieved the lowest or highest possible score. Psychometric properties (Internal consistency reliability, convergent validity, known-group validity, and construct validity) were assessed by descriptive and inferential statistics using SPSS version 26. Statistical significance was set as 0.05.

**Results:**

The mean age of the sample was 72.3 ± 6.1 years. The majority (56.8%) of the participants were females. No significant floor or ceiling effects were observed for any subscale of AAQ. Internal consistency reliability, measured by Cronbach’s alpha for psychosocial loss, psychological growth, and physical change subscales was 0.81, 0.77, and 0.74, respectively. Significant correlations were shown in Pearson’s correlation between all the AAQ subscales and WHOQOL-BREF domains, indicating the convergent validity. Known-group validity showed that married individuals and individuals diagnosed with chronic disease scored significantly higher mean on all three subscales of AAQ. The exploratory factor analysis extracted three factors with 57.77% cumulative variance with exceptions from the original version. Twenty-two items loaded to any of the factors except items 21and 23.

**Conclusion:**

The Sinhala version of the AAQ has sound psychometric characteristics and it is a culturally appropriate and reliable measure to assess attitudes towards aging among Sinhala-speaking older adults.

**Supplementary Information:**

The online version contains supplementary material available at 10.1186/s40359-025-03919-y.

## Introduction

Population aging refers to the increase in the number and percentage of the older population aged 60 years and above, and at the same time, a decrease in the number and percentage of the young population aged 15 years and below [1]. Although population aging began in high-income countries, currently, low and middle-income countries are also facing the same issue [2]. In Sri Lanka, there has been a rapid increase in the population over 60 years, and it is predicted that it will be 25% of Sri Lanka’s population by the year 2030 [3].

Multiple factors influence older adults’ mental health, including biological, psychological, social, and demographic characteristics. Life transitions, including retirement, changes in residence, and the death of a spouse or friend, also affect psychological health [4]. Physical and psychological status is affected by attitudes towards aging, which is a powerful predictor of health and mortality [5]. Further, cognitive, affective, and assessment components of behaviors regarding the process of aging as a personal experience are the components of attitudes towards one’s own aging [6].

Among older people, negative attitudes towards aging cause consequences for mental and physical health [7]. Attitudes towards aging are a comprehensive personal view of the experience of aging throughout life [8]. Older adults experience aging as just another stage of life, and they accept losses and changes as universal experiences due to longer lifespans [9]. It is much more important to determine the older adults’ attitudes towards aging, which may be beneficial to plan interventions to support the individuals in poorly addressed aspects [10].

The Attitudes to Aging Questionnaire (AAQ) is a widely used, psychometrically robust tool to measure attitudes towards aging, which has been specifically designed for use with older people [8]. The 24-item Attitudes to Aging Questionnaire (AAQ) consists of three subscales, namely: psychosocial loss, psychosocial growth, and physical change, providing a unique scale to measure successful aging. It facilitates the measurement of how individuals age across cultures, political-economic, and social circumstances [11]. Furthermore, AAQ was developed as a part of an international project on Quality Of Life (QOL) among elders collaboratively with WHO, with data from different cultures including Europe, North America, Japan, China, and Turkey [12]. Initially, the validity and reliability of the Attitudes to Aging Questionnaire have been tested by using an international data set [11].

Further, the translated Spanish version of the AAQ showed acceptable psychometric properties. Internal consistency reliability was ensured with Cronbach’s alpha for the subscales of the questionnaire: 0.59, 0.70, and 0.73. And 34% of the variance in the three-factor solution for factor analysis. Also, subscales have shown good construct validity with WHOQOL-BREF domains [13]. Regarding the cross-cultural validation of the AAQ to the Iranian population, it has been revealed that the Farsi version of AAQ showed favorable validity and reliability. It has reported Cronbach’s alpha coefficient between 0.85 and 0.93 for the subscales. Convergent validity is also proven, as there were associations between AAQ subscales with WHOQOL-BREF and SF-36 quality of life [14]. Further, this questionnaire has been validated in France, showing good internal consistency reliability with Cronbach’s alpha coefficient of 0.62 (Physical Change), 0.74 (Psychological Growth), and 0.75 (Psychosocial Loss). And good convergent validity has been shown with self-reported health and quality of life, showing prior expected associations [15].

Although it has been validated in different cultural contexts, there’s no validated Sinhala version currently available. Therefore, this study aimed to cross-culturally adapt and to evaluate the psychometric properties of the Sinhala version of the Attitudes to Aging Questionnaire to evaluate its suitability as a tool for assessing the attitudes towards aging among Sinhala-speaking older adults.

## Methods

### Study design, setting, and participants

This was a cross-sectional type study conducted among Sinhala-speaking older adults to validate the Sinhala version of the AAQ. A study was carried out among 185 older adults conversant in Sinhala in randomly selected residential care facility homes for elders in Colombo district, Sri Lanka. Participants from other ethnic backgrounds who were fluent in Sinhala were also eligible. Language proficiency was checked during recruitment to ensure that all participants could fully understand and respond to the questionnaire items.

Those older adults originally come from different areas of Sri Lanka, making it a diverse participant group. Further, institutionalized older adults across the country have similar socio-economic and cultural characteristics, as elder care institutions in Sri Lanka follow similar structures, admission criteria, and caregiving [16].

These older adults are confined to residential care facility homes primarily due to socioeconomic circumstances, including lack of caregivers, financial constraints, absence of a permanent home, or children residing abroad. They were not institutionalized for disease management or medical treatment. Therefore, the participants represented older adults living in residential care facility homes for elders are suitable for assessing attitudes toward their ageing.

Further, institutionalization is a growing trend in Sri Lanka, and residents in these settings often face social, psychological, and health-related challenges that may affect attitudes toward ageing. Thus, validating the Sinhala AAQ in this population was considered.

### Eligibility criteria

Participants were eligible for the study if they were aged 60 years or above, Sinhala-speaking, and residing in residential care facility homes for the elders. Individuals with severe cognitive impairment or acute illness that limited their ability to participate in the study were excluded.

Individuals who reported acute medical/surgical conditions, severe hearing impairment, or were diagnosed with psychiatric conditions were excluded.

### Sample and sample size calculation

A sample of older adults was recruited with a ratio of seven individuals per item, adhering to the rule of thumb of 5 to 10 subjects per item [17]. As the AAQ had 24 items, seven individuals per item were considered, and the initially calculated sample size was 168. After adding 10% for the non-respondents, the final sample size was calculated as 185.

### Sampling technique

Three residential care facility homes in the Colombo district were randomly selected using simple random sampling method (Lottery method). Then, ratios proportionate to the total number of elderly people in the randomly selected residential care facility homes were taken to achieve the sample of 185 participants.

### Cross-cultural adaptation of AAQ to the Sinhala language

The standard guidelines were followed for cross-cultural adaptation of the Sinhala version of AAQ [18]. The same method has been used in different tool validations related to older adults [19, 20]. The original English version of the AAQ was translated into the Sinhala language by two independent bilingual translators (Forward translation). One of the translators was a graduate nurse, and the other translator was a graduate in Linguistics. The two Sinhala translations were compared with the original English version of the questionnaire and an initial common translation. It was back translated into the English language by another two bilingual translators (a nursing academic, and a graduate from the arts stream) who have not accessed the original English version of the AAQ. Back translations were discussed among investigators, and a common Sinhala translation was produced. It was presented to an expert committee consisting of a consultant geriatrician, three nursing academics specialized in geriatric care, and two consultant community physicians. This expert committee reviewed the revised versions of AAQ for two rounds. Then, the pre-final version of the AAQ was developed, and the pre-final Sinhala version of the AAQ was pre-tested among 30 community-dwelling older adults and those who participated in the pilot testing were not included in the main validation sample. A pilot study was conducted with 30 older adults to assess clarity and comprehension of the Sinhala version. Feedback was used to make minor modifications. The 30 participants in the pilot study were not included in the main validation sample. The necessary modifications were made, and the final version of the Sinhala version of the AAQ was developed to assess its validity and reliability.

### Study instruments

The data needed for the validation process of the Sinhala version of the AAQ were collected by using a questionnaire containing the demographic characteristics, translated Sinhala version of the AAQ, along with the validated Sinhala version of the WHOQOL-BREF questionnaire. This tool was used to assess correlations for convergent and divergent validity of the subscales of AAQ, as the AAQ has been developed using the methodology for scale development collated by the WHO Quality of Life Group [13]. This method has been widely used by the previous validation studies of AAQ in different contexts, including Norway [21], Iran [14], and Spain [13].

### Attitudes to aging questionnaire

The original validation of the AAQ reported a three-factor structure, each subscale (Psychosocial loss, Physical change, and Psychological growth) consisting of eight items. Each item is scored on a 1–5 Likert scale, giving a subscale range of 8–40. Higher scores on the Physical Change and Psychological Growth domains indicate more positive attitudes towards aging, while higher scores on the Psychosocial Loss domain reflect more negative attitudes. Further, in the original validation study, only the separate scores for three subscales were obtained [11]. The AAQ was developed using the methodology of the World Health Organization Quality of Life Group and has been validated across different cultures. In the Spanish Version, Cronbach’s alpha for subscales ranged from 0.59 to 0.73, with 34% variance in the three-factor solution. Further, the Farsi Version of AAQ also has strong reliability, with a Cronbach’s alpha of 0.85–0.93 for its subscales.

### WHOQOL-BREF questionnaire

The Quality of Life BREF questionnaire is an international questionnaire initiated by the WHO. The WHOQOL-BREF instrument comprises 24 items, which measure the following broad domains: physical health, psychological health, social relationships, and environment [22]. There are two separate items to rate the overall quality of life and health status. Participants express how much they have experienced the items in the preceding 2 weeks on a 5-point Likert scale, which ranges from 1 (not at all) to 5 (completely). It has been validated in the Sinhala language [23].

### Data collection procedure

The data collection was done by the principal investigator following the obtaining of ethical approval from the Research Ethics Committee, Faculty of Medical Sciences, University of Sri Jayewardenepura (Ref. no. ERC 10/23).

The data collection was conducted at residential care facility homes after obtaining permission from the in-charge officers. Both verbal and written informed consent were obtained from each participant to ensure their understanding related to the study and volunteer participation. Further, data were collected respectfully and confidentially, ensuring the privacy and psychological comfort, as the study involved older adults who are considered a vulnerable population, and their right to withdraw from the interview at any time is protected. Following consent, AAQ was interviewer-administered as some participants had limited literacy or visual difficulties. To minimize potential interviewer bias, all the questionnaires were administered by the same investigator, and trained to administer the questionnaire to avoid influencing participants’ responses. The data collected was kept carefully and confidentially. The response rate was 100%.

### Data analysis

Data analysis was performed using the Statistical Package of Social Science version 26.0 (SPSS). The level of significance was considered as 0.05. Floor and ceiling effects were assessed for each AAQ subscale by calculating the proportion of participants scoring the lowest and highest possible values. if ≥ 15% of participants achieved the minimum or maximum score, it was considered the presence of a floor or ceiling effect. The internal consistency reliability of each domain of the Sinhala version of AAQ was examined by calculating Cronbach’s alpha coefficient separately, and the Cronbach’s alpha value for the overall scale was not calculated, adhering to the original validation of the AAQ, as the three subscales represent distinct constructs. An alpha value of at least 0.70 was considered the acceptable level of internal consistency reliability [15]. The validity of the AAQ was checked using convergent, known-group, and construct validity. Internal consistency reliability, convergent validity, and known group validity, Factor structure were initially assessed and reported using all 24 items of the AAQ as in the original tool. To assess the convergent validity, the association of the AAQ subscales with domains of WHOQOL-BREF was examined. Pearson correlation was performed to identify the associations. To assess the known group validity, the association of the AAQ subscales with socio-demographic variables was examined by performing an independent sample t-test. Exploratory factor analysis was performed to examine the factor structure of the AAQ. Kaiser–Meyer–Olkin index (KMO) and the Bartlett test of sphericity were performed to test if there was an underlying structure to the data. KMO > 0.40 was considered acceptable. Principal component analysis (PCA) with varimax rotation was used as the exploratory step to identify patterns in the data. Items exhibiting cross-loadings were assigned to the factor on which they had the highest loading, ensuring the conceptual relevance of each statement to the subscale. This approach has been commonly used in psychometric studies to guide factor interpretation and item assignment [24].

Further, confirmatory Factor Analysis (CFA) was performed to assess the factor structure. As there were very small latent variances observed in Factors 2 and 3, resulting in a non-positive definite covariance matrix, the full three-factor model could not be estimated by CFA. Considering these results, along with the conceptual relevance, the EFA-derived three-factor model was retained for the Sinhala version of the Attitudes to Aging Questionnaire.

## Results

### Socio-demographic characteristics of the sample

The study included 185 institutionalized older adults. Of the participants, 56.8% (105) were females. Mean (SD) age was 72.6 (6.58), and 65.9% aged between 60 and 74 years. Nearly half of the participants had only primary education or low (Table 1).

**Table 1:** Demographic characteristics of the study sample (*N* = 185)

Demographic characteristic		Frequency (%)
Gender	Female	105 (56.8)
	Male	80 (43.2)
Age group	60–74 years	122 (65.9)
	75yrs and above	63 (34.1)
Marital status	Married	137 (74.1)
	Single/ Widowed/ divorced/separated	48 (25.9)
Level of education	Primary education or low	95 (51.4)
	Secondary education or higher	90 (48.6)
Ethnicity	Sinhalese	159 (85.9)
	Tamil	19 (10.3)
	Muslim	7 (3.8)
Province of origin	Western	62 (33.5)
	southern	37 (20.0)
	Central	20 (10.8)
	Sabaragamuwa	28 (15.1)
	North western	18 (9.7)
	Other	20 (10.8)

### Floor and ceiling effect

Descriptive statistics indicated a variability in AAQ subscale scores. For Psychosocial Loss, scores ranged from 14 to 37 with a mean value of 31.9 (SD 4.7), Psychological Growth ranged from 17 to 38 (mean = 31.5, SD = 4.6), and for Physical Change from 20 to 39 (mean 32.1, SD = 4.0). All subscales showed slight negative skewness (-1.89, -1.22, and − 1.09, respectively).

Each subscale was examined for floor or ceiling effect (possible range 8–40). For the Psychosocial Loss subscale, the lowest observed score was 14 (1.1% of participants), and the highest was 37 (4.9%). For the Psychological Growth subscale, the lowest score was 17 (0.5%), and the highest was 38 (1.6%). For the Physical Change subscale, the lowest score was 20 (1.6%), and the highest was 39 (0.5%). No participants achieved the minimum (8) or maximum (40) on any subscale. As all percentages were below the 15% threshold [25], no significant floor or ceiling effects were detected.

### Internal consistency reliability

Cronbach’s alpha coefficient was reported as 0.811 for the psychosocial loss subscale, 0.772 for psychological growth, and 0.743 for physical change, reporting that internal consistency reliability is retained for all the subscales of AAQ (Table 2).

**Table 2:** Internal consistency reliability of the subscales of AAQ

Subscale	Mean	SD	Cronbach’s alpha
Psychosocial loss	31.93	4.65	0.811
Psychological growth	31.47	4.64	0.772
Physical change	32.11	3.99	0.743

### Convergent validity

AAQ psychosocial loss subscale showed a statistically significant positive correlation with WHOQOL-BREF physical domain (*r* = 0.803, *p* < 0.001) and psychological domain (*r* = 0.900, *p* < 0.001). Psychological growth subscale showed a statistically significant strong positive correlation with WHOQOL-BREF physical domain (*r* = 0.898, *p* < 0.001) and psychological domain (*r* = 0.855, *p* < 0.001), and a significant negative correlation with social relation (*r*=-0.322, *p* < 0.001) and environmental domain (*r*=-0.187, *p* = 0.011). Physical change subscale showed a statistically significant strong positive correlation with the physical (*r* = 0.938, *p* < 0.001) and psychological domain (*r* = 0.791, *p* < 0.001) of the WHOQOL-BREF scale. There was a significant negative correlation with the social relation domain (*r*=-0.160, *p* = 0.03) of the WHOQOL-BREF scale (Table 3).

**Table 3:** Convergent validity- association of the AAQ with WHOQOL- BREF domains

		Psychosocial loss		Psychological growth		Physical change	
		*r*	*p*	*r*	*p*	*r*	*p*
WHOQOL- BREF domains	Physical domain	0.803^**^	< 0.001	0.898^**^	< 0.001	0.938^**^	< 0.001
	Psychological domain	0.900^**^	< 0.001	0.855^**^	< 0.001	0.791^**^	< 0.001
	Social relation	0.059	0.425	-0.322^**^	< 0.001	-0.160^*^	0.030
	Environmental domain	0.014	0.846	-0.187^*^	0.011	-0.032	0.662

*r*: Pearson’s correlation coefficient *p*: *p*-value

^**^Correlation is significant at the 0.01 level (2-tailed)

^*^Correlation is significant at the 0.05 level (2-tailed)

### Known group validity

There was no difference between females and males for three subscales of AAQ (*p* > 0.05). Known group validity showed that married individuals and individuals diagnosed with chronic medical conditions scored significantly higher on the physical change, psychological growth, and psychosocial loss subscale (Table 4).

**Table 4:** Known group validity of the AAQ

		Psychosocial Loss		Psychological Growth		Physical Change	
		Mean (SD)	t Test, df, *p* value	Mean (SD)	t Test, df, *p* value	Mean (SD)	t Test, df, *p* value
Gender	Female	31.8 (4.51)	-0.133, 183, 0.894	31.4 (4.69)	-0.221, 183, 0.825	32.08 (3.81)	-0.129, 183, 0.897
	Male	31.9 (4.85)		31.5 (4.61)		32.16 (4.24)	
Marital status	Married	32.7 (3.6)	3.96, 183, **< 0.001**	32.16 (3.88)	3.48, 183, **< 0.001**	32.67 (3.50)	3.306, 183, 0.001
	Single/ Widowed/ divorced/separated	29.72 (6.36)		29.52 (5.97)		30.52 (4.83)	
Level of Education	Primary or lower	31.48 (5.16)	-1. 357, 183, 0.176	30.9 (5.19)	-1.724, 183, 0.086	31.67(4.28)	-1.563, 183, 0.120
	Secondary or higher	32.41(4.01)		32.07 (3.93)		32.58 (3.63)	
Diagnosed with chronic disease condition	Yes	32.95(2.88)	4.48, 183, **< 0.001**	32.19 (3.72)	3.06, 183, 0.003	32.83 (3.33)	3.56, 183, < 0.001
	No	29.86 (6.54)		30.01 (5.88)		30.67 (4.78)	

### Factor structure

The EFA, KMO was greater than 0.50 (KMO = 0.702 and the Bartlett test of Sphericity was significant (*p* < 0.001), indicating an underlying structure in the scale. Initially, six factors were extracted with the eigenvalue > 1. The Scree test and the parallel analysis suggested four or three factors. The four-factor solution produced one factor with only 2 items loaded for the 4th factor. So, the factor analysis was conducted using the extraction methods with a “fixed number of factors equal to 3. The first factor accounted for 39.08% of the variance, the second for 10.09%, and the third for 8.60%. Thus, three factors with a cumulative variance explained 57.77% were extracted with Principal Component analysis with Varimax rotation.

The three factors were named psychosocial loss, psychological growth, and physical change. Items loaded to a three-factor solution. There were different loadings across the subscales compared to the original tool.

In the pattern matrix of the three-factor solution, except for two items (Item 21 and 23), all the other items loaded ≥ 0.40 on any factor (Table 5). There were some deviations in loading the factors, including items 12 and 15 from the Psychosocial Loss subscale loaded on Physical Change and Psychological Growth, respectively. Items 8, 11, and 14 showed cross-loadings across multiple factors. In the Psychological Growth subscale, the items except 1, 2, and 5, other items loaded as in the original tool. The physical change subscale is differently loaded on the original scale. Only four items were loaded into this scale. Item 13 showed negative loading.

**Table 5:** Factors extracted by the exploratory factor analysis

Subscale as the original scale	Item no	Items	Factor 1 Psychosocial Loss	Factor 2 Psychological Growth	Factor 3 Physical Change
Psychosocial Loss	3	Old age is a time of loneliness.	0.821		
	6	Old age is a depressing time of life.	0.612		
	9	I find it more difficult to talk about my feelings as I get older.	0.691		
	12	I see old age mainly as a time of loss			− 0.491
	15	I am losing my physical independence as I get older.		0.870	
	17	As I get older, I find it more difficult to make new friends.	0.576		
	20	I don’t feel involved in society now that I am older.	0.676		
	22	I feel excluded from things because of my age.	0.509		
Psychological Growth	1	As people get older they are better able to cope with life			0.814
	2	It’s a privilege to grow old	0.714		
	4	Wisdom comes with age.		0.577	
	5	There are many pleasant things about growing older.	0.703		
	10	I am more accepting of myself as I have grown older.		0.797	
	18	It is very important to pass on the benefits of my experiences to younger people.		0.512	
	19	I believe my life has made a difference.		0.848	
	21	I want to give a good example to younger people.			
Physical Change	7	It is important to take exercise at any age.	0.828		
	8	Growing older has been easier than I thought.	0.598	0.541	
	11	I don’t feel old.	0.600	0.572	
	13	My identity is not defined by my age.	− 0.426		
	14	I have more energy now than I expected for my age.	0.633	0.511	0.430
	16	Problems with my physical health do not hold me back from doing what I want to.			0.759
	23	My health is better than I expected for my age.			
	24	I keep myself as fit and active as possible by exercising		0.866	

## Discussion

The current study evaluated the psychometric properties of the Sinhala version of the Attitudes to Aging Questionnaire. It was observed that the Sinhala version of AAQ has sound psychometric properties that are concordant with previous AAQ validation studies, where they have used the sample of older adults aged 60 years and older for the validation studies. Further mean age is concordant with the current study, including 71.1 years in Spain, 73.84 in French, 77.62 in Norwegian, and 72.32 in Canadian samples [13–15, 21]. The absence of significant floor or ceiling effects suggests that the AAQ has adequate sensitivity and is capable of discriminating the attitudes toward aging in this sample. Internal consistency reliability was assessed separately for each AAQ subscale and reported acceptable reliability of the Sinhala version of the AAQ, with higher reliability for psychosocial loss, psychological growth subscales, and lower reliability in the physical change subscale compared to the reliability of subscales reported in the first international study [11]. Furthermore, the current study revealed more acceptable reliability in all three subscales than the Spanish version [13] and the French version [15], but lower reliability than the Farsi version [14]. The possible reason may be that the statements of the AAQ are more sensitive to the translation procedure and the cultural context. Participants might have responded to statements related to general aspects of aging by considering their perspectives and experiences.

Convergent validity revealed that there were significant correlations between all three dimensions of AAQ and WHOQOL BREF domains, which is concordant with the previous validation studies, including Iran, Norwegian, and Spain contexts [13, 14, 21]. Concordant with the studies in other cultural contexts, the findings of the current study demonstrate a correlation between positive attitudes towards aging with positive physical, psychological, and social aspects of quality of life. Interestingly, the psychological loss dimension also showed a positive correlation with all QoL domains. Although psychological loss theoretically reflects negative attitudes toward ageing, this pattern may reflect cultural factors within the Sri Lankan context. Older adults who acknowledge age-related psychological or functional decline may demonstrate higher emotional adjustment and acceptance of ageing. Further, in relation to the known group validity, there were no gender differences for any subscale of AAQ (*p* > 0.05). This finding is contradictory compared to previously reported results in Spain, where they have reported significant differences in attitudes towards aging among males and females [13, 14]. Instead, marital status and diagnosis of chronic medical condition revealed a significantly higher mean for the subscales. The reason for these contradictory results may be due to the cultural background of the Sri Lankan older adults, where the perception regarding aging is similar among all individuals, despite their gender. But marital status may be a factor that affects their perception regarding aging, as in Sri Lankan culture, marriage has become a strong factor that affects the social and emotional health of the aging population.

Furthermore, the results of factor analysis showed a three-factor model accounting for 57.77% of the total variance. Even though the current study showed a three-factor model, exceptions were observed in item distribution for the three factors compared to the original scale. Items 21 and 23 did not load to any factor. This loading pattern shows some similarity with the Portuguese Version [26], where they also have observed the same feature of loading item 23.

Although items 8 and 11 belonged to the physical change subscale in the original version, the current study showed different loading with cross-loading loading those two items for both psychosocial loss and psychological growth subscales, with the highest loading on the psychosocial loss subscale. However, both items were aligned more closely with the psychological growth subscale than with psychosocial loss. Further, incorporating those two items into the psychological growth subscale with other loaded items contributed to increasing the internal consistency reliability of the subscale (Cronbach’s alpha = 0.915). So, it was decided to consider items 8 and 11 as items belonging to the psychological growth subscale. Although item 7, which belongs to the physical change subscale in the original tool has loaded to the Psychosocial loss subscale, conceptually it is highly aligned with the Physical change subscale. So, it was decided to retain it as in the original tool. Item 14, which belonged to the physical change subscale exhibited cross-loadings across all three subscales, with the lowest loading on its original subscale. But conceptually, this statement was more closely aligned with the physical change subscale, which supported the decisions to retain item 14 in the physical change subscale as in the original version [20]. Further, although items 2 and 5 loaded into the Psychosocial Loss subscale in the current study, those two items were retained with the Psychological Growth subscale as in the original tool because both items were conceptually more relevant to the Psychological Growth subscale. Item 13, which showed negative loading to the Psychosocial loss subscale was considered as an item in the Physical change subscale, as in the original AAQ, considering the conceptual relevance because it has shown an inverse relationship with the psychosocial loss [27].

Considering the factor structure, the Physical Change subscale had the least homogeneity as many of the items were loaded into the other two subscales. The reason may be due to the socio-demographic differences of the populations, where older adults might have interpreted some physical factors as psychological factors. Different factor loadings have been observed in the validation study conducted for the French version [15]. The variation in factor extraction observed in this study could be due to several reasons. Primarily, the attitude towards aging is greatly influenced by the cultural background, especially since Sri Lankans have specific cultural patterns closely bound to their psychological and social aspects.

This study has several limitations. This study aimed to culturally validate the AAQ among Sinhala-speaking older adults using an institutionalized sample confined to the Colombo district, Sri Lanka. Although the residents come from various areas across Sri Lanka, the residential care facility homes provide a heterogeneous sample, and socio-economic characteristics and care provision are similarly structured in residential care facilities across the country; this may not fully represent the broader older adult population, especially community-dwelling older adults in the country, which is a limitation.

In the current sample, more than one-third of participants were aged over 75 years; their literacy levels and cognitive ability could have influenced their understanding of certain questionnaire items. Although the questionnaire was administered by a trained investigator to minimize this potential bias, and the older adults who were diagnosed with psychiatric illness or cognitive impairment were excluded, there is a possibility of response bias due to varying levels of literacy and understanding among the older participants, which cannot be completely excluded. Further, interviewer administration may have introduced some degree of bias, although the measures were taken as described in the methods section to minimize such effects.

Although EFA supported a 3-factor structure for the Sinhala AAQ, the CFA for the full model could not be estimated. Relatively small sample size, which can limit the stability of covariance estimates may have contributed to this pattern, which is a limitation of the current study.

## Conclusion

The Sinhala version of the AAQ with twenty-two items has sound psychometric characteristics, including internal consistency reliability, convergent, known group validity, and factorability with alterations of factor distribution for three subscales compared to the original tool. Item distribution across subscales of the current version: Psychosocial Loss subscale contains 6 items (3,6,9,17,20,22), Psychological growth subscale expanded to 10 items (2,4,5,8,10,11,15,18,19,24), and Physical Change subscale contains 6 items (1,7, 12, 13, 14,16) with 22 items in the Sinhala version of the AAQ (Sinhala version of the AAQ is attached as a supplement). The current validated Sinhala version of AAQ can be used as a tool for assessing attitudes toward aging among Sinhala-speaking older adults. Further validation studies are recommended for other linguistic groups in Sri Lanka.

## Supplementary Information

Supplementary Material 1.

## Data Availability

The datasets used and/or analysed during the current study are available from the corresponding author upon reasonable request.

## Abbreviations

AAQ: Attitudes to Aging Questionnaire
KMO: Kaiser-Meyer-Olkin Measure of Sampling Adequacy
SPSS: Statistical package for the social sciences
SD: Standard deviation
WHOQOL: BREF-World Health Organization Quality of Life Instrument (Brief Version)
