# Supplementary material for: Cross-cultural adaptation and analysis of psychometric properties of the Sinhala version of the Attitudes to Aging Questionnaire for institutionalized older adults
Source: BMC Psychol. 2025 Dec 30;14:139. doi: 10.1186/s40359-025-03919-y (PMC12859880; doi:10.1186/s40359-025-03919-y)
Supplement: Supplementary file 1 — Supplementary Material 1. [file 40359_2025_3919_MOESM1_ESM.pdf]

වයස්ගත වීම පිළිබඳ ආකල්ප ඇගයීමේ ප්‍රශ්ණාවලිය

උපදෙස්

වයසට යාම පිළිබඳව, ඔබට දැනුණේ කෙසේද යන්න මෙම ප්‍රශ්ණාවලියෙන් අසනු ලබයි.
කරුණාකර සියලුම ප්‍රශ්න වලට පිළිතුරු සපයන්න. කිසියම් ප්‍රශ්නයක් සඳහා කුමන ප්‍රතිචාරය දිය යුතුදැයි ඔබට අවිනිශ්චිත නම්, වඩාත් ගැළපේ යැයි හැඟෙන ප්‍රතිචාරය තෝරන්න.
මෙය බොහෝවිට ඔබගේ පළමු ප්‍රතිචාරය විය හැක.

සැම විටම ඔබගේ ප්‍රමිතීන්, බලාපොරොත්තු,සතුට සහ ඔබ සැලකිලිමත් වන කරුණු පිළිබඳව සිතෙහි තබා ගන්න. ඔබගේ ජීවිතය පිළිබඳව ඔබ සාමාන්‍යයෙන් සිතන්නා වූ ආකාරය පිළිබඳව අප අසනු ලබයි.

නිදසුනක් ලෙසට, ඔබට සාමාන්‍යයෙන් වයසට යාම ගැන හැඟෙන ආකාරය පිළිබඳව, පහත පරිදි ප්‍රශ්නයක් අසන අවස්ථාවකදී, මම වයසට යෑමට අකමැතිය.

කිසිසේත්ම සත්‍යය නොවේ	සාමාන්‍යය වශයෙන් සත්‍යය වේ	මධ්‍යස්ථව සත්‍යය වේ	සැබැවින්ම සත්‍යය වේ	අතිශයින්ම සත්‍යය වේ
1	2	3	4	5

මෙහි සඳහන් ප්‍රකාශයන් ඔබට වඩාත් ගැළපෙන ආකාරය අනුව වඩාත් සුදුසුයැයි ඔබ තීරණය කරන්නා වූ පිළිතුරට හිමි අංකය තෝරා එය රවුම් කරන්න.වයසට යෑම පිළිබඳව ඔබට තෝරන්නේ "සැබැවින්ම සත්‍යයක් " හෙවත් 4 වන පිළිතුර නම් එය රවුම් කරන්න. එසේත් නැතිනම් ඔබ තෝරන්නේ "කිසිසේත්ම නැත" යන්න නම් අංක 1 රවුම් කරන්න. සැම ප්‍රශ්නයක්ම ඉතා හොඳින් කියවන්න. ඔබගේ හැඟීම් තක්සේරු කරන්න. ඔබට වඩාත් සුදුසුයැයි හැඟෙන පිළිතුර තෝරන්න.

පහත ප්‍රශ්න වලින් විමසනුයේ පහත සඳහන් ප්‍රකාශ සමඟ ඔබ <u>කෙතරම් දුරට එකඟ වනවාද</u> යන්නයි. ඔබ එකී ප්‍රකාශයන් සමඟ එකඟ වන්නේ නම් " අතිශයින්ම එකඟ වේ" යන්න ඉදිරියෙන් ඇති අංකයද, එකඟ නොවන්නේ නම් "කිසිසේත්ම එකඟ නොවේ" යන්න ඉදිරියෙන් ඇති අංකය ද රවුම් කරන්න. ඔබගේ පිළිතුර " අතිශයින්ම එකඟ වේ" හෝ " කිසිසේත්ම එකඟ නොවේ" යන්න අතර වේ නම් අදාළ පිළිතුර සහිත අංකය රවුම් කරන්න.

	කිසිසේත්ම එකඟ නොවේ	එකඟ නොවේ	අවිනිශ්චිතයි	එකඟ වේ	අතිශයින්ම එකඟ වේ
1. වයසට යාමත් සමග, මිනිස්සු වඩා හොඳින් ජීවිතයට මුහුණ දෙති.	1	2	3	4	5
2. වයස්ගත වීම වරප්‍රසාදයකි.	1	2	3	4	5
3. මහලු විය යනු, තනිකම දැනෙන කාලයකි.	1	2	3	4	5
4. වයස්ගත වන විට ප්‍රඥාවෙන් මුහුකුරා යයි.	1	2	3	4	5
5. වයසට යාම හා සබැඳි බොහෝ ප්‍රසන්න දේ තිබේ	1	2	3	4	5
6. මහලු විය යනු, ජීවිතයේ කණස්සල්ලට පත්වන කාලයකි.	1	2	3	4	5
7. ඕනෑම වයසකදී, ව්‍යායාම වල නිරත වීම වැදගත් වේ.	1	2	3	4	5

පහත ප්‍රශ්න, ඔබට පහත ප්‍රකාශ **කෙතරම්** සත්‍ය **දැයි** අසයි. ප්‍රකාශය ඔබට "අතිශයින්ම සත්‍ය නම්, අතිශයින්ම සත්‍යයක් වේ " අසල ඇති අංකය රවුම් කරන්න. ප්‍රකාශයන් ඔබට කිසිසේත්ම සත්‍ය නොවේ නම්, " කිසිසේත්ම සත්‍යය නොවේ " අසල ඇති අංකය රවුම් කරන්න. ඔබේ පිළිතුර "කිසිසේත්ම සත්‍යය නොවේ" සහ "අතිශයින්ම සත්‍ය වේ " අතර කොතැනක හෝ සඳහන් කිරීමට අවශ්‍ය නම් ඔබ ඒ අතර ඇති අංක වලින් එකක් රවුම් කළ යුතුය.

	කිසිසේත්ම සත්‍යය නොවේ	සාමාන්‍යය වශයෙන් සත්‍යය වේ	මධ්‍යස්ථව සත්‍යය වේ	සැබැවින්ම සත්‍යය වේ	අතිශයින්ම සත්‍යය වේ
8. වයස්ගත වීම මා සිතුවාට වඩා පහසු විය.	1	2	3	4	5
9. වයසට යාමත් සමග, මාගේ හැඟීම් පිළිබඳ කතා කිරීම ඉතා අපහසු බව මට හැඟේ.	1	2	3	4	5

10.වයස්ගත වීමත් සමඟම, මට මාව වඩාත් හොඳින් හඳුනා ගැනීමට හැකි වී ඇත.	1	2	3	4	5
11.මම මහලු වී ඇතැයි මට නොදැනේ.	1	2	3	4	5
12.මම දකින ආකාරයට, මහලුවිය ප්‍රධාන වශයෙන් අහිමි වීම් වලට මුහුණ දෙන කාලයකි.	1	2	3	4	5
13.මගේ අනන්‍යතාවය මගේ වයස මත තීරණය නොවේ.	1	2	3	4	5
14.මගේ වයසට අනුව, බලාපොරොත්තු වූවාට වඩා මා ශක්තිමත්ය.	1	2	3	4	5
15.වයස්ගත වෙත්ම, මාගේ කායික හැකියාවන් අඩු වෙමින් පවතී.	1	2	3	4	5
16.මට අවශ්‍ය දේ සිදු කිරීමට මගේ ශාරීරික සෞඛ්‍ය පිළිබඳ ගැටලු බාධාවක් නොවේ.	1	2	3	4	5
17.වයසට යාමත් සමග, මට අලුත් යහලුවන් ඇතිකර ගැනීමට අපහසු වී ඇත.	1	2	3	4	5
18.මගේ අත්දැකීම්වල ප්‍රතිලාභ තරුණ අයට ලබා දීම ඉතා වැදගත් වේ.	1	2	3	4	5
19.මගේ ජීවිතය විසින් යම් වෙනසක් සිදුකර ඇති බව මම විශ්වාස කරමි	1	2	3	4	5
20.වයස්ගත වීමත් සමඟ පෙර මෙන් මට දැන් සමාජයට සම්බන්ධ බවක් නොදැනේ.	1	2	3	4	5
21.මගේ වයස නිසා මාව බොහෝ දෙයින් ඈත් වී ඇති බව මට හැඟේ.	1	2	3	4	5
22.හැකි සෑම විටම, ව්‍යායාම තුලින් ශක්තිමත්ව හා ක්‍රියාශීලීව සිටීමට මම උත්සාහ දරනවා.	1	2	3	4	5

ඔබ ලබා දුන් සහයෝගයට බොහොම ස්තුතියි
